# Potential prognostic determinants for *FET::CREB* fusion-positive intracranial mesenchymal tumor

**DOI:** 10.1186/s40478-024-01721-2

**Published:** 2024-01-30

**Authors:** Frank M. Mezzacappa, Frankie K. Smith, Weiwei Zhang, Andrew Gard, Fatmagul Kusku Cabuk, Ignancio Gonzalez-Gomez, Hector L. Monforte, Jiancong Liang, Omkar Singh, Martha M. Quezado, Kenneth D. Aldape, Murat Gokden, Julia A. Bridge, Jie Chen

**Affiliations:** 1 Department of Neurological Surgery, University of Nebraska Medical Center, Omaha, NE USA; 2 Department of Pathology, Microbiology, and Immunology, University of Nebraska Medical Center, 983135 Nebraska Medical Center, Omaha, NE 68198 USA; 3 Department of Pathology, University of Utah and Huntsman Cancer Institute, Salt Lake City, UT USA; 4 Department of Neurological Surgery, MD West ONE, Omaha, NE USA; 5 Department of Pathology, Basaksehir Cam and Sakura City Hospital, Başakşehir, Turkey; 6 Division of Pathology, Johns Hopkins All Children’s Hospital, Petersburg, FL USA; 7 Department of Pathology, Microbiology, and Immunology, Vanderbilt University Medical Center, Nashville, TN USA; 8 Laboratory of Pathology, National Cancer Institute, National Institutes of Health, Bethesda, MD USA; 9 Department of Pathology, University of Arkansas Medical Center, Little Rock, AR USA; 10 Division of Molecular Pathology, ProPath, Dallas, TX USA

**Keywords:** Intracranial mesenchymal tumor, *FET::CREB* fusion, Fluorescence in situ hybridization, Next-generation sequencing, Genome-wide DNA methylation profiling

## Abstract

Intracranial mesenchymal tumor (IMT), *FET::CREB* fusion-positive is a provisional tumor type in the 2021 WHO classification of central nervous system tumors with limited information available. Herein, we describe five new IMT cases from four females and one male with three harboring an *EWSR1::CREM* fusion and two featuring an *EWSR1::ATF1* fusion. Uniform manifold approximation and projection of DNA methylation array data placed two cases to the methylation class “IMT, subclass B”, one to “meningioma-benign” and one to “meningioma-intermediate”. A literature review identified 74 cases of IMTs (current five cases included) with a median age of 23 years (range 4–79 years) and a slight female predominance (female/male ratio = 1.55). Among the confirmed fusions, 25 (33.8%) featured an *EWSR1::ATF1* fusion, 24 (32.4%) *EWSR1::CREB1*, 23 (31.1%) *EWSR1::CREM*, one (1.4%) *FUS::CREM*, and one (1.4%) *EWSR1::CREB3L3*. Among 66 patients with follow-up information available (median: 17 months; range: 1–158 months), 26 (39.4%) experienced progression/recurrences (median 10.5 months; range 0–120 months). Ultimately, three patients died of disease, all of whom underwent a subtotal resection for an *EWSR1::ATF1* fusion-positive tumor. Outcome analysis revealed subtotal resection as an independent factor associated with a significantly shorter progression free survival (PFS; median: 12 months) compared with gross total resection (median: 60 months; *p* < 0.001). A younger age (< 14 years) was associated with a shorter PFS (median: 9 months) compared with an older age (median: 49 months; *p* < 0.05). Infratentorial location was associated with a shorter overall survival compared with supratentorial (*p* < 0.05). In addition, the *EWSR1::ATF1* fusion appeared to be associated with a shorter overall survival compared with the other fusions (*p* < 0.05). In conclusion, IMT is a locally aggressive tumor with a high recurrence rate. Potential risk factors include subtotal resection, younger age, infratentorial location, and possibly *EWSR1::ATF1* fusion. Larger case series are needed to better define prognostic determinants in these tumors.

## Introduction

Intracranial mesenchymal tumor (IMT), *FET::CREB* fusion-positive is a provisional tumor type in the 2021 World Health Organization (WHO) classification of central nervous system tumors [27]. It represents primary intracranial mesenchymal neoplasms that typically affect children and young adults. These tumors are characterized by the fusion of a *FET* RNA-binding protein family gene, which includes Ewing sarcoma RNA binding protein 1 (*EWSR1*) and fused in sarcoma (*FUS*), to a cAMP response element-binding protein (*CREB*) family gene, which includes activating transcriptase factor-1 (*ATF1*), cAMP responsive element binding protein 1 (*CREB1*), cAMP response element modulator (*CREM*), and cAMP responsive element binding protein 3 like 3 (*CREB3L3*). IMTs are rare tumors with only scattered case reports and small case series published in the literature [35, 36, 40], so specific information regarding their characteristics, especially their clinical behavior, is not well known. In the current study, the clinicohistopathologic and molecular findings of five new cases of *FET::CREB* fusion-positive IMT are detailed. Additionally, a comprehensive review of the literature to summarize relevant clinical, histopathological, genetic, treatment, and outcome information of this unique tumor type is reported.

## Materials and methods

### Tumor sample collection

Five IMT cases with documented *FET::CREB* fusion were collected from participating institutions. Fusions were confirmed by either fluorescence in situ hybridization or next-generation sequencing at referring laboratories. In addition, four of the cases were subjected to methylation profiling. The following patient characteristics and clinical data were retrieved from hospital records: age, sex, presenting symptoms, MRI characteristics, tumor location and size, extent of resection, histology, fusion partners, methylation profile, treatment, and follow-up data.

### Fluorescence in situ hybridization

Fluorescence in situ hybridization (FISH) analysis was performed on representative unstained 5-μm formalin-fixed paraffin-embedded (FFPE) sections of cases 1, 2, 3, and 5 using an *EWSR1* break-apart probe set (Agilent Technologies, Santa Clara, CA) followed by reflex studies with three fusion probe sets in the following order: *EWSR1::ATF1*, *EWSR1::CREB1*, and/or *EWSR1::CREM*. The three custom fusion probe sets utilized BAC clone cocktails that were selected based on their location in the UCSC Human Genome Browser (http://genome.ucsc.edu) and were obtained from the BACPAC sources of BACPAC Genomics (https://bacpacresources.org). Probes were directly labeled by nick translation and hybridized as previously described [1]. Each clone was also hybridized to normal metaphases to confirm correct mapping, optimal signal intensity, and lack of cross-hybridization. Results were evaluated using the thresholds established by in-house validation studies.

### Next-generation sequencing

Nucleic acid was extracted from representative unstained 5-μm FFPE sections of Case 4 and subjected to a solid tumor panel for sequence analyses of 238 genes and a fusion panel targeting over 700 exons of 117 cancer genes at the Children’s Hospital of Philadelphia (CHOP). Extracted DNA was fragmented and tagged using SureSelect^QXT^ target enrichment to generate adapter-tagged libraries. Biotin-labeled probes specific to the targeted regions were used for capture hybridization. Libraries were enriched for the desired regions using streptavidin beads and then subjected to sequence analysis on Illumina MiSeq or HiSeq platform for 150 bp paired-end reads. RNA sequencing libraries were prepared using Archer Universal RNA Reagent Kit with CHOP fusion panel custom-designed primers with target specific molecular barcode. Sequencing data were analyzed using Archer™ Analysis for fusion genes.

### Genome-wide DNA methylation profiling

Genome-wide methylation profiling was performed on cases 1, 2, 3, and 4. Briefly, genomic DNA was extracted from FFPE tissue sections after macro-dissection to enrich for viable tumor content using the All Prep DNA/RNA FFPE kit (Qiagen). The DNA was bisulfite converted using EZ DNA Methylation Kit (Zymo Research D5001) and subsequently processed using Infinium Methylation EPIC 850K kit according to the manufacture’s protocol (Illumina). The beadchips were scanned on iScan reader (Illumina) and output idat files were processed through the DKFZ CNS classifier versions v11b6 and v12b6 (publicly available, unpublished) of the CNS tumor methylation classifier [8]. In addition, we requested the National Cancer Institute Laboratory of Pathology to provide a dataset of ~ 7500 brain tumors with 198 classes. Raw idat files were processed using single sample noob normalization available in minfi R package [5]. Prior to this we removed any probes with detection *p* value less than 0.05. Because this given dataset of ~ 7500 samples consists of mixed data from the 450k and EPIC arrays, we selected common probes (n = 452,453) between the two arrays. In the next step, we removed sets of probes that consisted of probes on X and Y chromosomes, single nucleotide polymorphism related probes, and probes not uniquely mapped to human reference genomes. After filtering of probes, 357,483 common probes were selected on the EPIC/450k array for further analysis. We performed unsupervised clustering on all these samples and calculated 198 principal components with most variable 20k probes and used these data to create uniform manifold approximation and projection (UMAP).

### Literature search

A comprehensive review of the literature was performed to identify all reported cases of IMT with confirmed *FET::CREB* fusion up to September 2023 in the English literature. Specifically, PubMed was queried with the search terms “intracranial mesenchymal tumor AND fusion”, “angiomatoid fibrous histiocytoma AND intracranial”, and “(intracranial mesenchymal tumor OR angiomatoid fibrous histiocytoma) AND fusion”. All case reports, case series, and review articles that presented new cases of IMT with *FET::CREB* fusion were included. Additionally, references for all articles were reviewed to evaluate for any further cases that were not revealed in the initial literature search. All available clinical, pathological, genetic, treatment, and outcomes information were extracted for each individual patient. Reports that did not specify both fusion partners were excluded from further analyses.

### Statistical analyses

The Kaplan–Meier method was used to estimate the probability of survival. Progression-free survival (PFS) and overall survival (OS) were analyzed for extent of resection (EOR), age, tumor location, and fusion partner. PFS was defined as the time between initial diagnosis and radiographic recurrence or last follow-up. OS was defined as the time from initial diagnosis to death. For each factor, the Log-rank test was performed to compare the survival times among the groups. Cox proportional hazard regression models were used for multivariate analysis. R packages “survival” and “survminer” and GraphPad prism version 9.5.1 (GraphPad Software, San Diego, CA, USA) were used to perform the analyses. A *p*-value less than 0.05 was considered statistically significant.

## Results

### Case presentations

#### Clinical characteristics

The median age for the five new cases of IMT with confirmed *FET::CREB* fusion in the current series was 26 years old (range 13–32 years; Table 1). There were four female patients and one male patient. Four of the patients (Cases 2–5) presented with symptoms associated with elevated intracranial pressure such as headache and changes to vision. Patient 1 presented with a new onset generalized tonic–clonic seizure. None of the patients had a history of previous CNS disease or any previous chemoradiation therapies for other disease processes.

**Table 1:** Clinical, pathological, treatment, and outcome information for the five new IMT cases

	Age (years)	Sex	Presentation	Tumor location	Imaging	Initial surgical management	Histology	Gene fusion	Outcome
Case 1	26	F	Generalized tonic–clonic seizure	Anterior Falx	Avidly enhancing lobulated 1.7 × 1.3 × 1.1 cm mass with surrounding vasogenic edema	GTR	Hypercellular areas composed of spindled cells in whorls. Hypocellular areas composed of spindled or epithelioid cells in small nests or cords in a myxoid background. A dense lymphocytic infiltrate at the periphery as well as within the tumor	*EWSR1::CREM*	Recurrence free at 12 months
Case 2	27	F	Headache and blurry vision; papilledema	Atrium of right lateral ventricle	Homogenously enhancing 2.3 × 3.0 × 3.6 cm mass with obstructive hydrocephalus	GTR	Densely packed groups of cells in a collagenous stroma, or singly distributed cells in a myxoid background. Abundant lymphocytes, plasma cells, and scattered histiocytes	*EWSR1::ATF1*	Recurrence free at 6 months
Case 3	32	M	Headache	Inferior right frontal lobe near midline	Heterogeneously enhancing 3.5 cm mass	GTR	Predominantly epithelioid cells with pleomorphic, large, hyperchromatic nuclei, intranuclear inclusions and abundant cytoplasm. Some cells are spindled. Mixed inflammatory cells, plasma cells, multinuclear giant cells, and histiocytes	*EWSR1::CREM*	Recurrence free at 1 month
Case 4	17	F	Headache and neck pain; papilledema	Left frontal, superior paramedian, involving middle and posterior frontal lobe	Solid and cystic mass with prominent adjacent edema and midline shift	GTR	Hypercellular, tightly packed sheets of small round tumor cells with scant cytoplasm. Scattered mitoses and apoptotic debris. No lymphocytic infiltrate	*EWSR1::CREM*	Recurrence at 3 months treated with proton beam radiotherapy; later developed bilateral pulmonary metastases and treated with chemotherapy; alive with extensive disease at 27 months
Case 5	13	F	Intermittent blurry vision; papilledema	Suprasellar	Heterogeneously enhancing solid and cystic mass measuring 2 cm with compression of the optic chiasm	Needle biopsy	Scattered spindled cells in a myxoid stroma. No mitotic activity	*EWSR1::ATF1*	Developed profound cerebral edema at 8 months; recurrence free at 2 years

#### Imaging characteristics

**Fig. 1:**
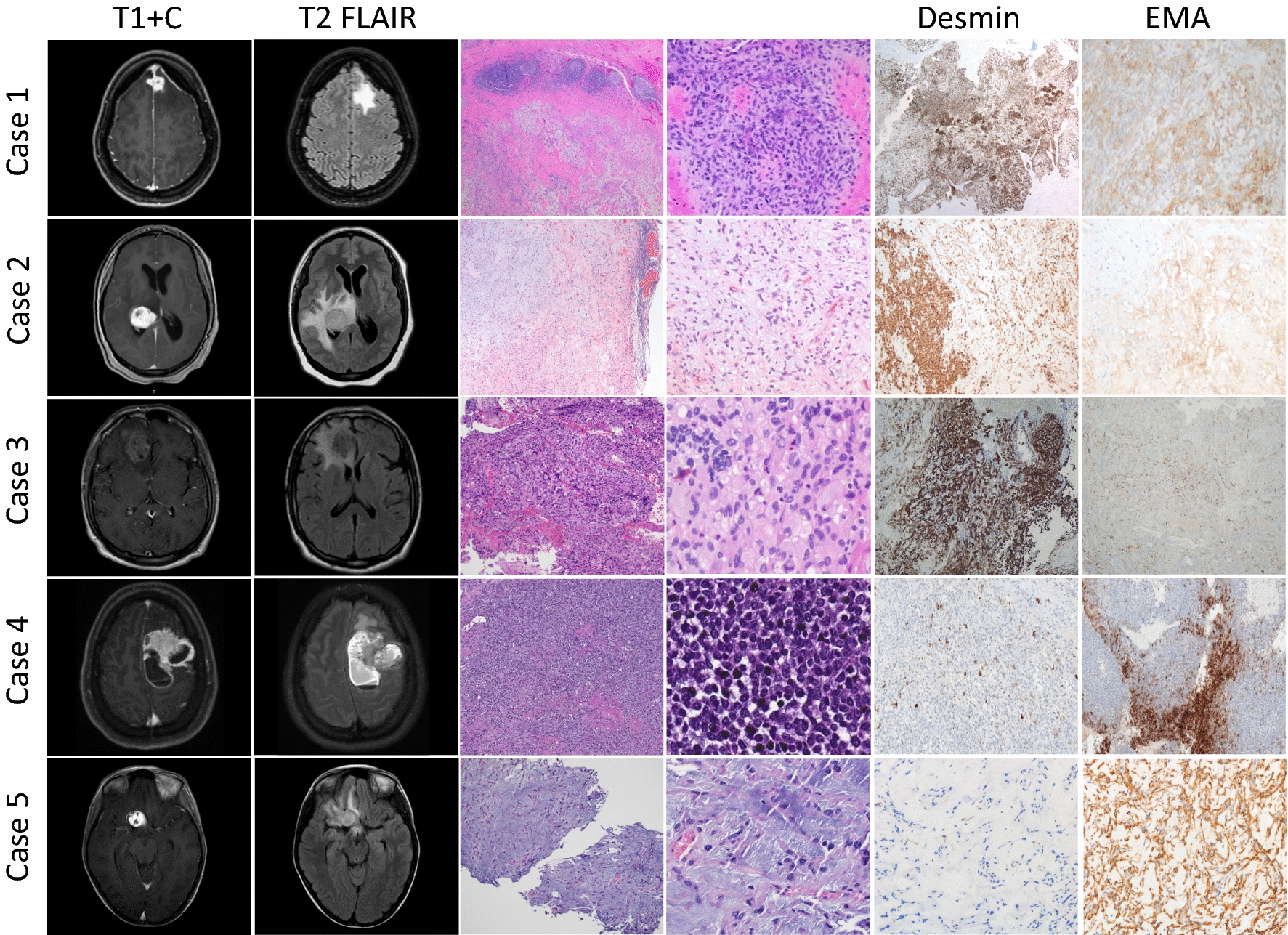
Representative images of MRI T1 post contrast (T1 + C), T2 FLAIR, H&E, and immunohistochemical stains for Cases 1–5. On MRI imaging, all five tumors were supratentorial, well-circumscribed, avidly enhancing lesions with surrounding vasogenic edema; however, on H&E stained sections, they showed heterogenous morphology. Cases 1 and 2 were composed of spindled/stellate tumor cells with a dense lymphocytic infiltrate, while Cases 3 and 4 featured sheets of epithelioid cells and highly-packed small round blue cells, respectively. Case 5 was distinct for its relatively low cellularity and a prominent myxoid stroma. By immunohistochemistry, desmin was positive in all cases except Case 5, and all five cases showed at least focal positivity for EMA

All five patients presented with supratentorial lesions (Fig. 1). Three tumors (Cases 1, 3, and 4) were localized to the frontal lobe, one (Case 2) was intraventricular, and the final tumor (Case 5) was in the suprasellar space. All tumors were well-circumscribed, avidly enhancing with surrounding vasogenic edema. Two tumors (Cases 4 and 5) showed both solid and cystic components.

#### Histological and immunohistochemical characteristics

The five tumors demonstrated heterogenous histological findings (Fig. 1). Cases 1 and 2 were well-circumscribed, lobulated lesions with a dense lymphocytic infiltrate at the periphery as well as within the tumor. The spindled/stellate tumor cells were arranged in whorls, small nests, or cords in a myxoid or collagenous stroma. Case 3 was composed of sheets of epithelioid cells with significant nuclear pleomorphism. A mixed lymphoplasmacytic inflammatory infiltrate was again noted. Case 4 consisted of hypercellular, tightly-packed small round cells with scattered karyorrhectic debris. A significant lymphocytic infiltrate was not appreciated. Case 5 exhibited scattered spindle cells in a prominent myxoid stroma. Mitotic activity and Ki-67 labelling indices were low in four cases but the Ki-67 labelling index was elevated to 40% in the hypercellular areas in Case 4.

By immunohistochemistry, all five cases showed at least focal positivity for EMA, CD99, and vimentin. Desmin was positive in four cases (diffuse in Cases 1–3 and focal in Case 4) and was negative in only one case (Case 5). Synaptophysin was focally positive in two cases. Focal positivity for CD68, MUC4, GLUT1, and S100 was observed in one case each. The hypercellular, primitive-appearing areas in Case 4 were positive for CD56, AE1/AE3, and SOX9, but were negative for synaptophysin and NKX2.2. PAS stain only highlighted occasional cells with cytoplasmic glycogen deposits, which disappeared after diastase treatment. All five tumors were negative for SSTR2, progesterone receptor, STAT6, Olig2, GFAP, Cam5.2, and smooth muscle antigen.

#### Genetic and epigenetic characteristics

**Fig. 2:**
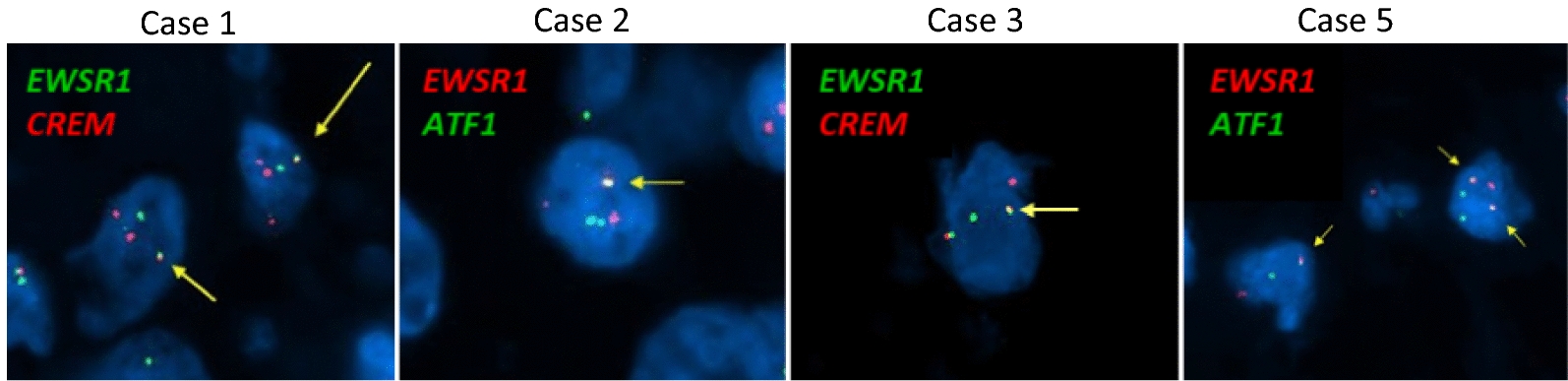
Representative images of fluorescence in situ hybridization for Cases 1, 2, 3, and 5. Yellow arrows indicate fusions. *EWSR1::CREM* fusion was detected in Cases 1 and 3 and *EWSR1::ATF1* in Cases 2 and 5

**Fig. 3:**
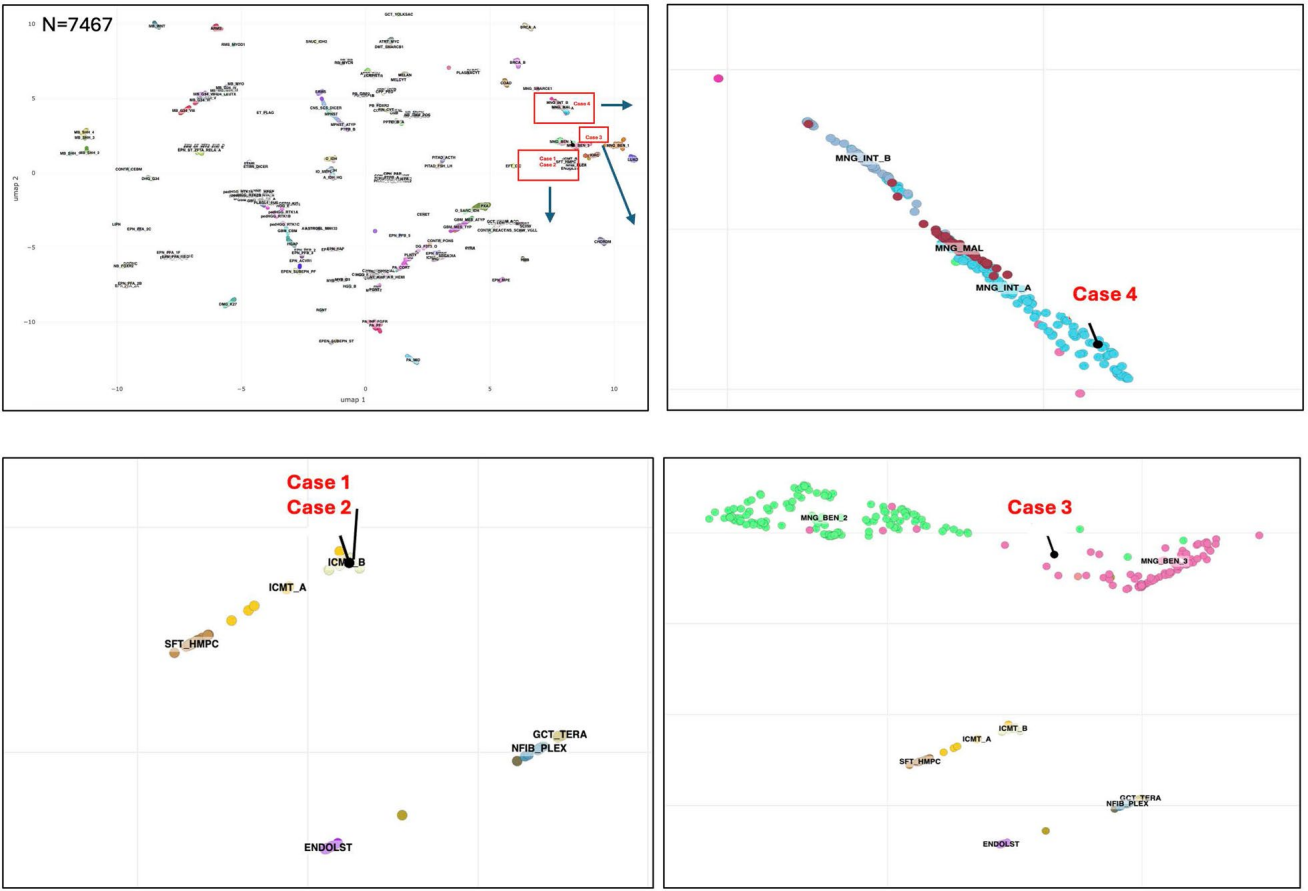
UMAP embedding of DNA methylation array data for Cases 1, 2, 3, and 4. Unsupervised clustering was performed on four samples using the NCI reference set (n = 7467) consisting of 198 classes. Cases 1 and 2 were placed to the methylation class “intracranial mesenchymal tumor subclass B (ICMT_B)”, Case 3 to “meningioma subclass benign_3 (MNG_BEN_3)”, and Case 4 to “meningioma subclass intermediate_A (MNG_INT_A)”

FISH analysis, performed on four cases, identified the following fusion events: *EWSR1::CREM* (Cases 1 and 3) and *EWSR1::ATF1* (Cases 2 and 5; Fig. 2). Next generation sequencing was performed on Case 4 and identified an *EWSR1::CREM* fusion. Four cases (Cases 1, 2, 3, and 4) had adequate tissue for DNA Methylation profiling. UMAP embedding of DNA methylation array data placed Cases 1 and 2 to the methylation class “intracranial mesenchymal tumor subclass B (ICMT_B)”, Case 3 to “meningioma subclass benign_3 (MNG_BEN_3)”, and Case 4 to “meningioma subclass intermediate_A (MNG_INT_A)” (Fig. 3). When analyzed on the DKFZ classifier, Case 3 matched to “MNG_BEN_3 with low scores (0.68 on version v11b6 and 0.34 on v12b6) while Case 4 was unclassifiable on both versions.

#### Treatment strategies and outcomes

Four patients, Cases 1–4, underwent surgical resection with gross total resection (GTR) achieved in all of them. None of those four patients received any adjuvant therapies after initial resection. The patients in Cases 1–3 were alive and neurologically intact without evidence of disease at their most recent follow-up (follow-up ranging from 1 to 12 months). The patient in Case 4 experienced tumor recurrence at 3 months after initial resection, which was treated with proton beam radiotherapy. Her tumor further progressed and she was found to have multiple bilateral pulmonary metastases, which were treated with adjuvant chemotherapy. She remains alive 27 months after the initial resection with extensive disease.

Patient 5 was initially thought to have a glioma after identification of a suprasellar mass on imaging and was empirically treated with vincristine and carboplatin. After tumor progression, she underwent a stereotactic needle biopsy, which revealed a *FET::CREB* fusion-positive IMT, and received proton beam radiotherapy. Eight months after the biopsy, she developed profound cerebral edema that culminated in a state of cerebral herniation. An emergent right-sided hemicraniectomy was performed. She continued to have edema despite being treated with steroids, therefor another resection was performed, which revealed necrotic tissue without evidence of tumor. The MRI scan at 2 years post biopsy showed no evidence of recurrence.

### Literature search

A comprehensive literature review identified 74 cases (to include the current 5 cases) of IMT with confirmed *FET::CREB* fusion reported in the English literature [3, 6, 7, 10–26, 31–36, 38–45]. There were an additional eight cases with reported *EWSR1* rearrangement but lacked identification of the fusion partner; therefore, these were not included in the analysis [2, 4, 8, 9, 28, 30]. Most prior publication represent case reports. A few small case series have been published recently [35, 36, 40], the largest containing 20 cases [35, 36].

#### Clinical features

**Fig. 4:**
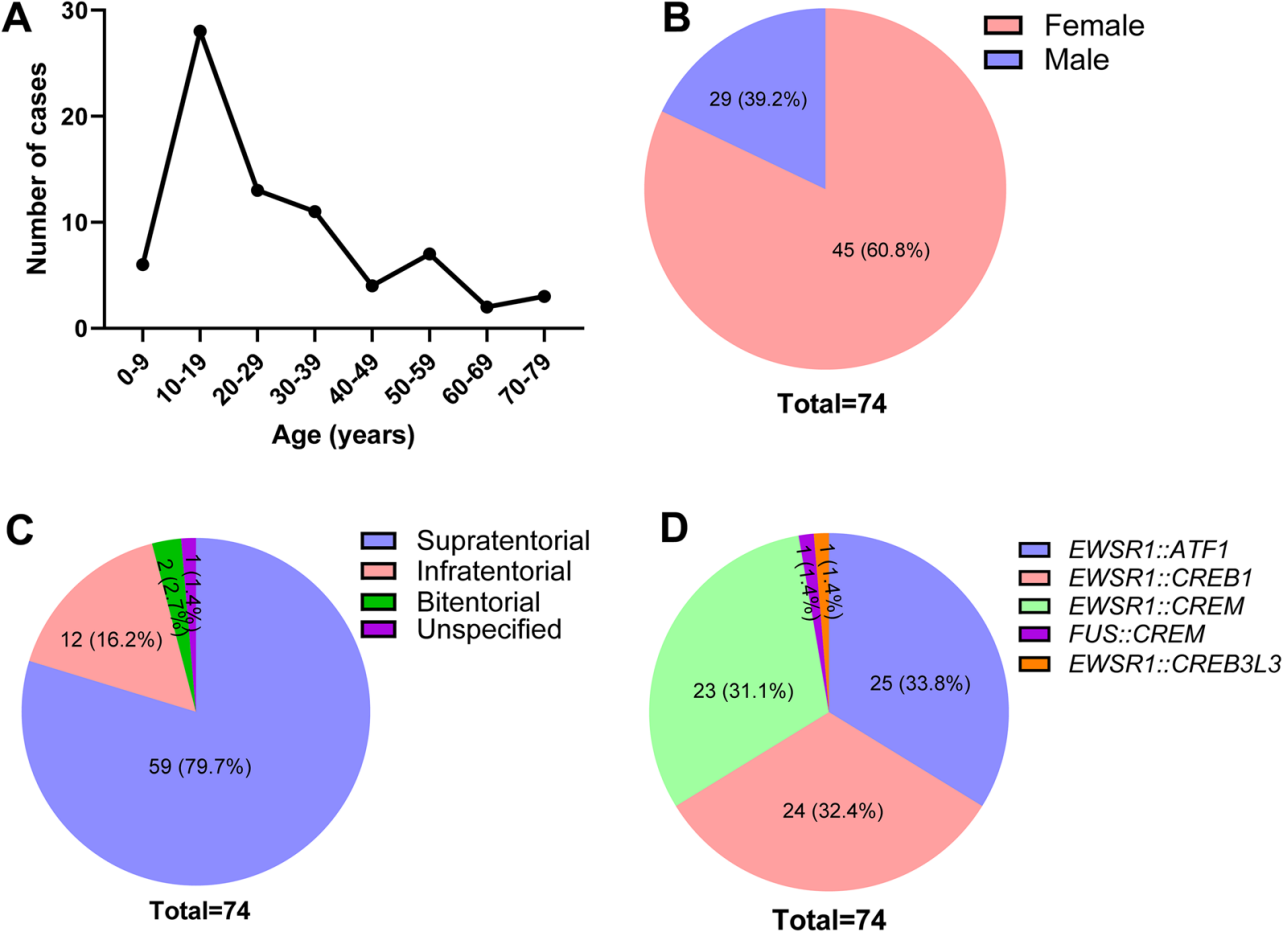
Age, sex, location, and fusion partners of the 74 cases of IMT reported to date with the five new cases included. The tumors predominantly occurred in children and young adults (median age 23 years old; **A** with a female predominance (F:M = 1.56; **B** Fifty-nine (79.7%) tumors were supratentorial, 12 (16.2%) were infratentorial, two (2.7%) were at the tentorium with both supratentorial and infratentorial extension, and the location of the remaining one (1.4%) was unclear (**C**). Among the 74 tumors with documented fusions, 25 (33.8%) featured an *EWSR1::ATF1* fusion, 24 (32.4%) *EWSR1::CREB1*, 23 (31.1%) *EWSR1::CREM*, one (1.4%) *EWSR1::CREB3L3*, and the last one (1.4%) *FUS::CREM* fusion (**D**)

The tumors can occur in any age but predominantly in children and young adults (median age 23 years old; range 4–79 years old; Fig. 4A) with a female predominance (female 45, male 29; F/M ratio = 1.56; Fig. 4B). Presenting symptoms were available for 40 (55%) patients and were extremely variable, the most common being signs of elevated intracranial pressure such as headache, nausea, vomiting, and changes to vision. Other common symptoms included seizure, new cranial nerve deficit, and altered mental status.

#### Imaging features

Among the 74 tumors, 59 (79.7%) were supratentorial, 12 (16.2%) were infratentorial, two (2.7%) were at the tentorium with both supratentorial and infratentorial extension, and the location of the remaining one (1.4%) was unclear (Fig. 4C). Furthermore, 44 (59.5%) tumors were extra-axial, 13 (17.6%) were intraventricular, 12 (16.2%) were intraparenchymal, three (4.1%) were in the spinal column, and two (2.7%) were sellar or parasellar. On imaging, these tumors appear predominantly circumscribed, contrast-enhancing lesions with frequent cystic components and surrounding vasogenic edema.

#### Histopathological and immunohistochemical features

Histologically, these tumors are frequently well-circumscribed with a multinodular appearance. Many tumors demonstrate a dense lymphoplasmacytic cuffing at the periphery, some with germinal center formation. Intratumoral lymphoplasmacytic infiltrates are also commonly observed. The tumor cells can be spindled/stellate, epithelioid/rhabdoid, or have a primitive appearance with a high nuclear/cytoplasmic ratio. The stroma can be myxoid or collagenous. Mitotic activity is usually low, but brisk mitotic activity has been reported in the literature and is also observed in our Case 4.

By immunohistochemistry, the tumor cells are frequently positive for EMA, desmin, and CD99, either diffusely or focally. Positivity for vimentin, CD68, S100, MUC4 are variable. SSTR2A, Olig2, GFAP, STAT6, and CD34 are usually negative.

#### Genetic and epigenetic features

These tumors are characterized by the fusion of a *FET* family gene member, most commonly *EWSR1* and rarely *FUS*, to a *CREB* family gene member, which includes *ATF1*, *CREB1*, *CREM*, and *CREB3L3*. This molecular hallmark can usually be confirmed by FISH or DNA/RNA sequencing. Among the 74 tumors with documented fusions, 25 (33.8%) featured an *EWSR1::ATF1* fusion, 24 (32.4%) *EWSR1::CREB1*, 23 (31.1%) *EWSR1::CREM*, and one (1.4%) *EWSR1::CREB3L 3* [29]. There has only been one (1.4%) *FUS::CREM* fusion [35] reported (Fig. 4D).

Information regarding the epigenetic features of IMT, *FET::CREB* fusion-positive are limited. Among 35 tumors with methylation data available, most cases (28/35; 80%) were not classifiable using the DKFZ CNS or sarcoma classifiers at the time of publication. Three cases from Sloan et al.’s cohort matched to the methylation class “AFH” on the DKFZ sarcoma classifier version 12.2 [35, 36], although one case from Tauziede-Espariat et al.’s cohort closely approximated “AFH” [40].

#### Treatment strategies

Surgical resection was attempted initially for all cases of the current study with the exception of Case 5, where chemotherapy was initially applied followed by the biopsy and then proton radiation. GTR was achieved in 42 of the 73 (57.5%) cases, with subtotal resection (STR) in 19 (26%) and degree of resection not specified in 11 (15.1%). Most patients did not receive adjuvant chemoradiation therapies, but eight of the 74 patients (10.8%) underwent radiotherapy following initial resection (6 STR and 2 GTR), one (1.4%) underwent chemotherapy after STR, and six (8.1%) underwent combination radiation and chemotherapy after STR or GTR of a tumor with aggressive histology.

#### Outcomes

**Fig. 5:**
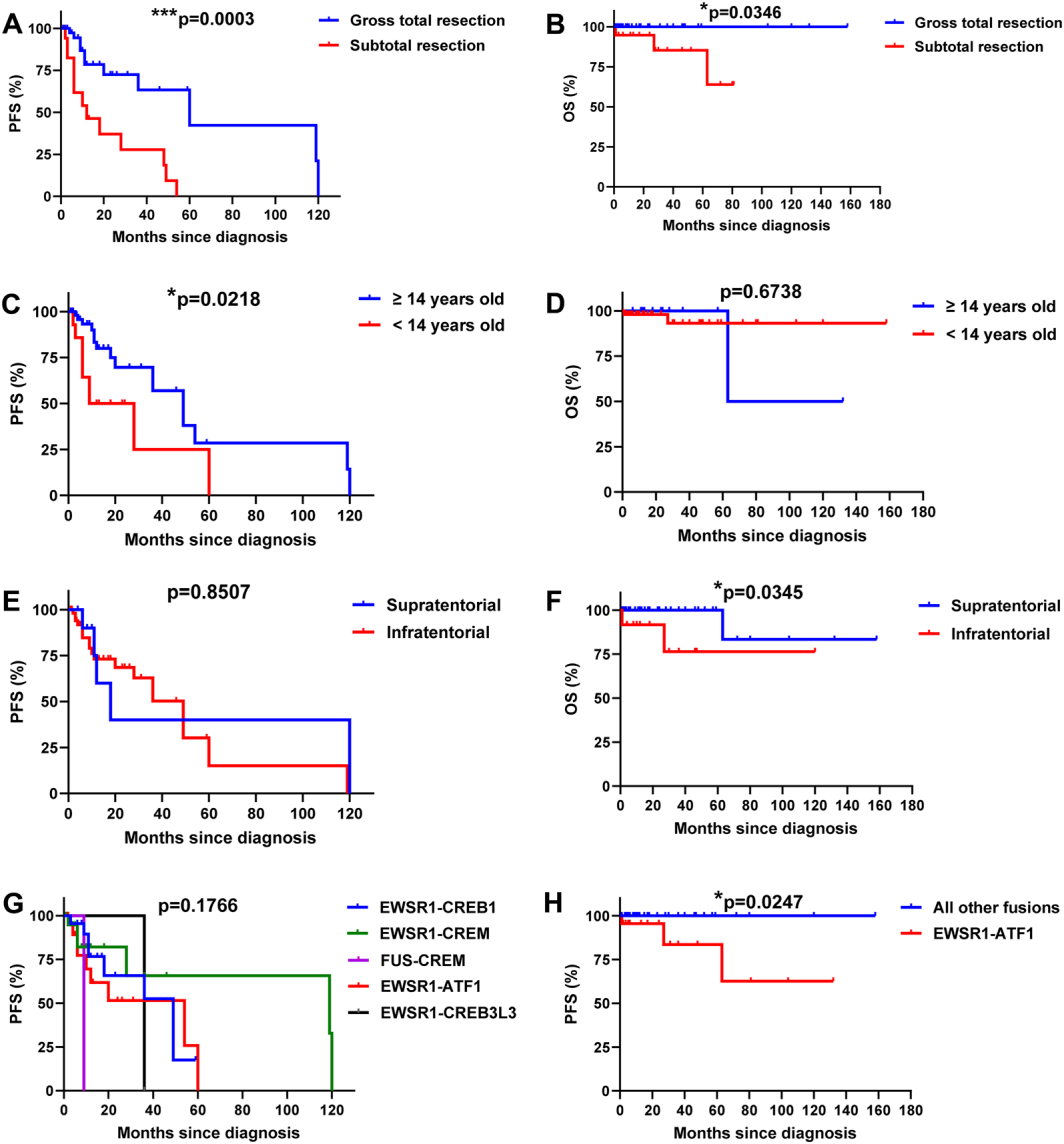
Kaplan–Meier analysis of the 66 IMT cases with available outcome data based on extent of resection, age, tumor location, and fusion partners. **A**, **B** Patients who underwent subtotal resection (STR) had a significantly shorter PFS and OS compared with patients who underwent gross total resection (GTR; *p* = 0.0003 and 0.0346, respectively). **C**, **D** Patients younger than 14 years old showed a shorter PFS (*p* = 0.0218) but not OS (*p* = 6738) compared with patients of or older than 14 years. **E**, **F** Patients with infratentorial tumors did not demonstrate a shorter PFS (*p* = 0.8507) but did show a shorter OS (*p* = 0.0345) compared with patients with supratentorial tumors. **G**, **H** No significant difference in PFS (*p* = 0.1766) was observed among tumors with different fusion partners; however, patients with *EWSR1::ATF1* fused tumors had a shorter OS (*p* = 0.0247) compared with patients with tumors harboring other fusions

Of the 74 patients, 66 (89.2%) had outcome data available with a median follow-up period of 18 months (range: 1–158 months). Of these 66, 26 (39.4%) patients experienced progression/recurrences after initial surgery (median 10.5 months; range 0–120 months; Fig. 5). Of the 26 patients with recurrences, 14 (53.8%) had STR and 11 (42.3%) had GTR. The extent of resection of the remaining one (3.8%) was unclear. Furthermore, 10 of these 26 (38.5%) patients had a tumor with an *EWSR1::ATF1* fusion, eight (30.8%) had *EWSR1::CREB1* fusion, six (23.1%) had *EWSR1::CREM,* one (3.8%) had *EWSR1::CREB3L3*, and one (3.8%) had *FUS::CREM* fusion. Three of the 66 patients (4.5%) patients eventually died at 1, 27, and 63 months after initial surgery. One was a 9-year-old girl with a frontal tumor, and the other two were a 17-year-old girl and a 70-year-old man with cerebellopontine angle tumors. All three patients underwent STR of IMTs harboring an *EWSR1::ATF1* fusion.

We further assessed the impact of different clinicopathologic parameters on patient survival. We first evaluated the effect of the extent of the resection (GTR and STR) on prognosis. Among 59 patients with known extent of resection and outcome, 40 patients underwent GTR and 19 patients underwent STR. Patients who underwent STR had a significantly shorter PFS (median: 12.0 months) compared with patients who underwent GTR (median: 60 months; *p* = 0.0003; Fig. 5A). STR also led to a significantly shorter OS (median: 60 months) compared with GTR (median not reached; *p* = 0.0346; Fig. 5A, B).

Next, we evaluated the effect of age on prognosis. Among 66 patients with known age and outcome, 14 (21.2%) were younger than 14 years, who showed a shorter PFS compared with patients of or older than 14 years (median 18.5 vs. 49 months, respectively; *p* = 0.0218); however, OS was not significantly different among these two groups (*p* = 0.6331; Fig. 5C, D).

We then evaluated the effect of tumor location on prognosis. Among 64 patients with a known tumor location and outcome, 12 (18.8%) had infratentorial tumors and 52 (78.8%) had supratentorial tumors. Although there was no significant difference in the median PFS among these two groups (18 months vs. 49 months, respectively; *p* = 0.8507), patients with infratentorial tumors demonstrated a shorter OS compared with patients with supratentorial tumors (*p* = 0.0345; Fig. 5E, F). We further analyzed whether the effect of tumor location on prognosis was dependent on EOR or age. Of the 12 patients with infratentorial tumors, seven underwent GTR, four underwent STR, and the EOR in the remaining one was unclear. Patients with infratentorial tumors who underwent STR seemed to have a shorter PFS (9 months) than patients who underwent GTR (120 months); however, this difference was not statistically significant (*p* = 0.055). No significant difference was observed in OS between these two groups (*p* = 0.1). Among the 52 patients with supratentorial tumors, 32 underwent GTR, 14 underwent STR, and the EOR in the remaining six was unclear. Patients with supratentorial tumors who underwent STR had a shorter PFS (10 months) compared with patients who underwent GTR (60 months; *p* = 0.0017), although no significant difference was observed in OS between the two groups (*p* = 0.32). No significant difference was observed between the ages of patients with infratentorial tumors and supratentorial tumors (22 vs. 23 years old; *p* = 0.7866), either.

Next, we evaluated the effect of different fusion partners on prognosis. Among 66 patients with known outcomes, no significant difference in PFS (*p* = 0.1766) was observed among tumors with different fusion partners; however, patients with *EWSR1::ATF1* fused tumors had a shorter OS compared with patients with tumors harboring other fusions (*p* = 0.0247; Fig. 5G, H).

**Fig. 6:**
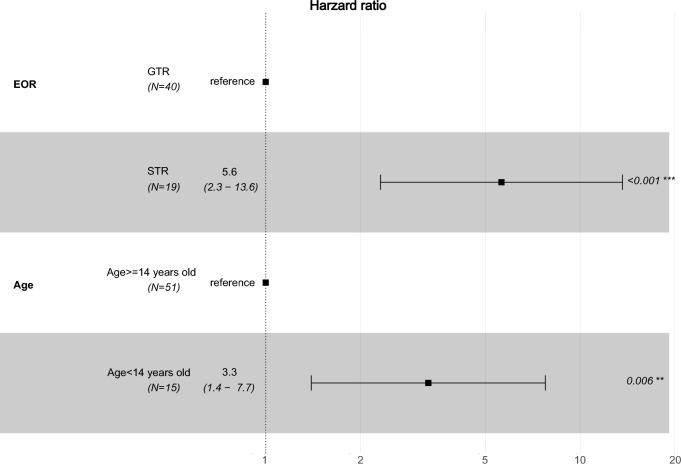
Multivariate Cox regression analysis of the 66 IMT cases with available outcome data. Subtotal resection (STR; HR: 5.6, confidence interval: 2.3–13.6, *p* < 0.001) and a young age < 14 years (HR: 3.3, confidence interval: 1.4–7.7, *p* = 0.006) remained as independent risk factors leading to a shorter progression free survival

Finally, we performed multivariable Cox regression analysis to evaluate the effects of all the above parameters on survival (Fig. 6). STR (HR: 5.6, confidence interval: 2.3–13.6, *p* < 0.001) and a young age < 14 years (HR: 3.3, confidence interval: 1.4–7.7, *p* = 0.006) remained as independent risk factors leading to a shorter PFS, while tumor location (HR: 1.54, confidence interval: 0.52–4.55, *p* = 0.43) and fusion partners (HR: 1.56, confidence interval: 0.64–3.85, *p* = 0.32) did not (data not shown).

## Discussion

The 2021 WHO classification of tumors of the central nervous system included a new provisional tumor type termed “intracranial mesenchymal tumor, *FET::CREB* fusion-positive” [27]. These tumors are rare and aspects regarding the potential breadth of clinical behavior are not well-known. In the current study, detailed clinicopathological and molecular findings of five novel cases of IMT, *FET::CREB* fusion-positive are reported. In addition, an extensive literature review identified 69 additional published cases of IMT, *FET::CREB* fusion-positive. The majority of these cases occurred in children and young adults with a slight female predominance. Evaluation of the clinical outcome of these patients revealed STR, younger age (< 14 years old), infratentorial location, and possible *EWSR1::ATF1* fusion as poor prognostic factors for this recently defined tumor type.

The five new cases of *FET::CREB* fusion-positive IMT in the current study occurred in adolescence to early adulthood and showed a wide morphologic spectrum, from spindled/stellate cells of various cellularity (Cases 1, 2 and 5), to sheets of epithelioid cells in Case 3, and to highly-packed small blue cells with frequent mitoses in Case 4. The patients’ outcomes in the current cases were also variable, with patients 1–3 showing a stable course after GTR while patient 4 suffering an early recurrence at 3 months and later developing bilateral pulmonary metastases. These clinicopathologic features are consistent with those previously described in the literature [35, 36, 40].

In recent years, genome-wide DNA methylation profiling has emerged as a powerful tool for CNS tumor classification. Tauziede-Espariat et al. [40] and Sloan et al. [36] were the first two groups to utilize DNA methylation profiling to study IMT, *FET::CREB* fusion-positive. Among the 11 cases reported by Tauziede-Espariat et al. [40], none of them was classifiable using DKFZ brain tumor version 11b4 or sarcoma classifier version 12.2 at publication, although one closely approximated the methylation class of extra-CNS “AFH”, one “clear cell sarcoma”, and two “solitary fibrous tumors”. Similarly, Sloan et al. [36] reported that only three of their 20 IMTs aligned with the methylation class “AFH” on the DKFZ sarcoma classifier version 12.2 with a calibrated score of greater than 0.9, indicating a high confidence classification. The remaining 17 cases did not reliably classify as “AFH” or any other class on the sarcoma classifier or the CNS tumor classifier version 11b4. However, their 20 cases did resolve into two distinct epigenetic subgroups that were both divergent from all other intracranial tumors and soft tissue sarcomas. In the current cohort of five new IMT cases, four had sufficient tissue for methylation profiling and UMAP embedding analysis. Among those, two cases (Cases 1 and 2) were placed to the methylation class “intracranial mesenchymal tumor”, while the other two cases were placed to different subclasses of meningioma (Case 3 to “benign_3” and Case 4 to “intermediate_A”), which is very intriguing. To the best of our knowledge, no cases in the literature have been classified in a meningioma category before. Although IMTs show distinct genetic alterations from meningiomas, both tumor types frequently present as dural-based lesions with EMA immunoreactivity. In addition, the tumor in Case 4 eventually metastasized to bilateral lungs, which are a common extra-CNS site of metastasis for meningiomas [37]. Taken together, the possibility of a shared cell of origin between IMT and meningioma cannot be completely excluded. In addition, the presence of a *FET::CREB* fusion itself may not be enough to place a tumor to the methylation class “intracranial mesenchymal tumor”, as what happened to Cases 3 and 4 in our cohort. To further investigate this possibility, we compared the two tumors (Cases 1 and 2) that were placed to the methylation class “intracranial mesenchymal tumor” by UMAP embedding and the two tumors (Cases 3 and 4) that did not. The morphology of Cases 1 and 2, spindled/stellate tumor cells arranged in whorls, small nests, or cords in a myxoid or collagenous stroma, was similar in appearance to AFH. In contrast, the majority of tumor cells in Case 3 were epithelioid with significant nuclear pleomorphism and Case 4 featured hypercellular, tightly-packed small round cells with scattered karyorrhectic debris. It is possible that some genetic alterations other than the *FET::CREB* fusion have led to the divergent morphological and epigenetic phenotypes of Cases 3 and 4. Further studies with larger cohorts are necessary to explore the full epigenetic spectrum of IMT, *FET::CREB* fusion-positive and its relationship to AFH, clear cell sarcoma, solitary fibrous tumor, meningioma, and the other tumors.

To date, little is known about the prognostic factors for patients with IMT, *FET::CREB* fusion-positive. Sloan et al. tried to analyze the effects of EOR and mucin-rich versus mucin-poor stroma on prognosis [35]. However, due to limited numbers of cases (20 cases in their own cohort plus 18 cases from the literature review) included in their analysis, no statistically significant difference was observed, although STR seemed to be associated with an increased risk of death and tumor recurrence. Here, with a larger cohort of 74 patients (5 new cases and 69 cases from the literature review), we identified EOR as a significant prognostic factor for IMT, *FET::CREB* fusion-positive. Kaplan–Meier analysis revealed that STR led to significantly shorter PFS and OS compared with GTR. Multivariable Cox regression analysis further confirmed STR as an independent prognostic factor associated with both inferior PFS and OS. Our results suggested that GTR should be achieved whenever possible in patients with IMT, *FET::CREB* fusion-positive for the best outcome.

In addition to EOR, our study identified age as a significant prognostic factor for IMT, *FET::CREB* fusion-positive. Patients younger than 14 years old had a significantly shorter PFS compared with patients of 14 years or older. Multivariable Cox regression analysis again confirmed a younger age as an independent risk factor associated with inferior PFS. Our results are consistent with what Sloan et al. reported on their epigenetic classification of IMT, *FET::CREB* fusion-positive [36]. In their study, they analyzed their cohort of 20 patients by genome-wide DNA methylation array profiling and identified two distinct epigenetic subgroups. They found that Group B tumors, which occurred most often in early childhood (median age 7 years, range 4–15 years) had an inferior PFS relative to Group A tumors, which occurred frequently in adolescence or early adulthood (median age 15 years).

Our study also suggested *EWSR1::ATF1* fusion as a possible prognostic factor for IMT, *FET::CREB* fusion-positive. Among the 74 IMT cases reported to date, three patients eventually died of disease, all of whom underwent a STR for an *EWSR1::ATF1* fused tumor. Sloan et al. analyzed patient survival (OS and PFS) stratified by fusion type (*EWSR1::CREB1*, *EWSR1::CREM*, and *EWSR1::ATF1*) but did not find a statistically significant difference [35]. In their later analysis with epigenetic data, they found that Group A, which had a favorable outcome, contained mostly *EWSR1::ATF1* and *EWSR1-CREB1* fusions, while Group B composed of more *CREM* fused tumors (either *EWSR1::CREM* or *FUS::CREM*) [36]. Here, we report that although *EWSR1::ATF1* fusion did not impact PFS, tumors with *EWSR1::ATF1* fusion did show a statistically shorter OS when compared with tumors harboring other fusions. Since we still only have three patients who died of disease, this finding needs to be interpreted with caution. Further study with a larger cohort is necessary to evaluate the precise prognostic effect of different fusion types.

Besides EOR, age, and *EWSR1::ATF1* fusion, we further identified another novel prognostic factor: tumor location. Although most IMT cases arose in the supratentorial areas, infratentorial tumors demonstrated shorter OS compared with their supratentorial counterparts by Kaplan–Meier analysis. However, no significant difference was observed in PFS.

In conclusion, the findings of the current study confirm that IMT, *FET::CREB* fusion-positive is a locally aggressive tumor with a high recurrence rate (~ 40%). The results also suggest that IMT, *FET::CREB* fusion-positive can be risk-stratified by several basic clinicopathologic parameters. Potential risk factors include subtotal resection, younger age, infratentorial location, and possibly *EWSR1::ATF1* fusion. Larger case series are needed to better define prognostic determinants in this unique tumor type.

## Data Availability

All data generated or analyzed during this study are included in this published article.

## Abbreviations

ATF1: Activating transcriptase factor-1
CREB: CAMP response element-binding protein
CREM: CAMP response element modulator
EOR: Extent of resection
EWSR1: Ewing sarcoma RNA binding protein 1
FISH: Fluorescence in situ hybridization
FUS: Fused in sarcoma
GTR: Gross total resection
IMT: Intracranial mesenchymal tumor
OS: Overall survival
PFS: Progression-free survival
STR: Subtotal resection
WHO: World Health Organization
